# Statistical shape analysis of hand and wrist in paediatric population on radiographs

**DOI:** 10.3906/sag-2002-176

**Published:** 2020-08-26

**Authors:** Ural KOÇ, İlker ERCAN, Senem ÖZDEMİR, Semih BOLU, Ayşegül YABACI, Onur TAYDAŞ

**Affiliations:** 1 Department of Radiology, Ankara Şehit Ahmet Özsoy State Hospital, Ankara Turkey; 2 Department of Biostatistics, Faculty of Medicine, Uludağ University, Bursa Turkey; 3 Department of Anatomy, Faculty of Medicine, Uludağ University, Bursa Turkey; 4 Department of Pediatric Endocrinology, Adıyaman Training and Research Hospital, Adıyaman Turkey; 5 Department of Biostatistics and Medical Informatics, Faculty of Medicine, Bezmialem Vakıf University, İstanbul Turkey; 6 Department of Radiology, Faculty of Medicine, Sakarya University, Sakarya Turkey

**Keywords:** Pediatrics, bone development, bone age, morphometrics, hand and wrist

## Abstract

**Background/aim:**

The goal of this study was to compare differences in hand and wrist shapes and to evaluate these according to growth and allometry in children on radiographs related to bone age.

**Materials and methods:**

The study included 263 males and 189 females. A total of 452 left hand and wrist radiographs were retrospectively collected. Standard anatomical landmarks marked on radiographs.

**Results:**

There were seen to be significant differences in comparisons of hand and wrist shapes according to sex (P = 0.009). The most suitable model in the growth models was seen as the Gompertz growth model for both females and males (model P < 0.001). For the relationship between shape and size to evaluate allometry, significant models were obtained in females (model P = 0.017, MSE = 0.0002) and in males (model P < 0.001, MSE = 0.0002). In our study, the difference between the sexes was found mostly in the radiocarpal region. It was observed that the deformation of the carpal bones started in the distal row carpal bones.

**Conclusion:**

Significant differences were found in hand and wrist shapes according to sex. Models for growth and allometry of hand and wrist shapes were found to be significant in children.

## 1. Introduction

Growth refers to the increase in body volume and mass, and development refers to the acquisition of biological functions. As an individual grows from foetal life through childhood and puberty, and completes growth as a young adult, the skeletal bones change in shape and size. One of the best criteria for the evaluation of growth and development is the determination of the maturity of the bones. Skeletal maturity, also known as “bone age”, is a key indicator for biological maturity and indicates progress towards total fusion of the epiphyses of the long bones [1–3]. Bone age is determined with evaluation of the distal radius, distal ulna, carpal and metacarpal bones on left-hand wrist radiographs according to various atlases [1–3]. Sex and racial differences also need to be taken into consideration for skeletal maturation [4].

A significant innovation in analysing skeletal maturation is concerned with the way in which shape and size are characterised [5,6]. New methodologies and concepts, which are known collectively as geometric morphometrics, are being increasingly used in evolutionary contexts, since they make it possible to distinguish between a component of size change over time (growth) and one of shape change over time (development). Most of the studies in medicine are concerned with the examination of geometric properties of anatomical structure [7]. Qualitative or quantitative datasets in the statistical analysis have commonly consisted of measuring values, and today’s anatomical structure shape or appearance has begun to be used as input data with the development in imaging techniques [5–9].

Geometric morphometrics (GM) is a tool used for statistical analysis of shape based on Cartesian landmark coordinates. Geometric morphometric methods utilise a mathematical definition of shape. Shape comprises all the features of landmark configuration other than overall size, position, and orientation. In geometric morphometrics, the “real” size of a specimen is shown by the size of each landmark configuration, which is captured as its ‘centroid size’ (CS). CS is a scalar accounting for the actual distance (in the actual scale of each specimen) between the landmarks and the centroid of the configuration (the geodesic centre of the configuration). The most common measure of size utilised in GM is centroid size (CS), which is the square root of the summed square distances between all the landmarks and their centroid [8–12].

The relationship between size and shape is called “allometry”. This refers to the size-related changes in morphological traits and remains a fundamental concept for the study of development and evolution [10,11,13,14]. Allometry is an example of the biological state in which deformation (shape variance) is associated with size changes. For allometric variation, the process which is considered to produce these correlated effects is growth. It is emphasised that different growth rates in parts of an organism cause different forms of the organism, so allometry is very significant for the evolution of morphology and shape [15]. There are two different forms of allometry, namely, static and ontogenetic. Ontogenetic allometry is defined as the change in shape associated with age or stage of development, while static allometry refers to variation in shape among individuals at a given age or stage [16]. For the majority of anatomical structures, proportions and shapes have a regular relationship with size. Many genetic and environmental factors are effective on growth and it is important to understand the extent of allometric variability [10–16]. In our study, deformation related to age or developmental stage was evaluated for the hand and wrist using the geometric morphometric method.

The goal of this study was to compare differences in hand and wrist shapes and to evaluate these according to growth and allometry in children on radiographs related to bone age.

## 2. Materials and methods

### 2.1. Subjects

Cases presenting at the Paediatric Endocrinology outpatient clinic of Adıyaman Training and Research Hospital between January and September 2017 were screened and 452 left hand and wrist radiographs were collected in total. The study included 263 males with a median age of 10 years (range, 1–18 years) and 189 females with a median age of 11 years (range, 2–17 years). The preliminary diagnosis for wrist radiograph were short height in 230 cases (50.88%), obesity in 118 (26.11%), premature adrenarche in 21 (4.65%), premature thelarche in 20 (4.42%), precocious puberty in 15 (3.32%), gynecomastia in 9 (1.99%), hypothyroid in 8 (1.77%), general examination in 7 (1.55%), retarded development in 6 (1.33%), and hirsutism, congenital adrenal hyperplasia, tall height, primary amenorrhea, type 1 diabetes mellitus, adrenal failure, coeliac disease, goitre, hypertrichosis or micropenis in the remaining 18 (3.96%). Approval for the study was given by the Local Ethics Committee (2017/13/08) and all procedures were applied in conformity with the Declaration of Helsinki. We waived the informed consent due to the retrospective nature of the study.

### 2.2. The estimation of bone age

Bone age evaluation was made by 2 radiologists with 7 and 8 years of experience due to create ground truth bone age values regarding the normalization of images for study population, by accepting the Gilsanz and Ratib (GR) atlas as reference, with no knowledge of the calendar age of the cases and no consultation with each other. After these evaluations, any cases of disagreement were evaluated again by the radiologists together and consensus was reached on bone age. The consistency of the radiologists was evaluated with the intraclass correlation coefficient (ICC). The evaluations of both radiologists were found to be consistent (intraclass correlation coefficient [ICC] = 0.99, P < 0.001).  

### 2.3. Acquisition of images

The conventional left-hand wrist radiographs were obtained using a Rotanode device (Toshiba, Tokyo, Japan) in posteroanterior projection with 55 kV voltage and exposure of 5 mAs (there could be variability depending on the age of the patient) at a distance of 1 meter, with the centre point determined as the midmetacarpal region of the left hand.

### 2.4. Collection of two-dimensional bone landmarks

**Figure 1:**
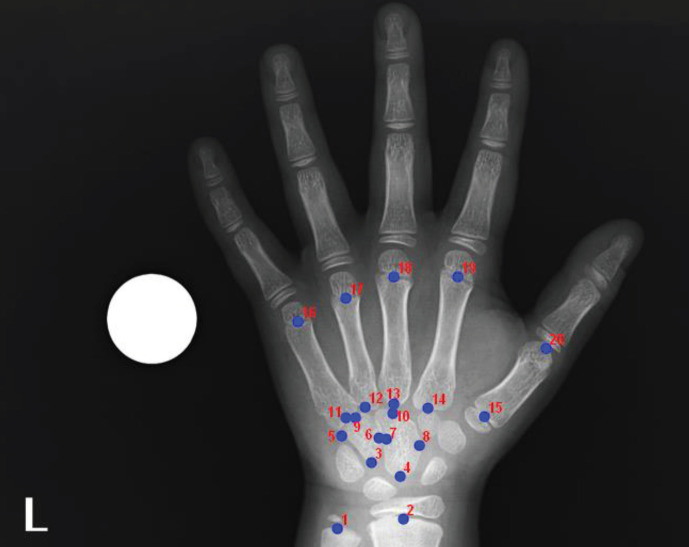
Landmarks (1-20) on the left-hand radiography used in this study were shown.

The data of the hand wrist radiographs were collected from 2-dimensional (2D) digital images. The anatomical landmarks were identified and marked by a single radiologist using TpsDig version 2.30 software. A total of 20 landmarks were marked on the digital hand wrist radiographs loaded onto the software (Figure 1). The descriptions of the landmarks are given in Table.

**Table:** Definitions of landmarks used in present study.

Number	Landmarks
1	Midline point of epiphyseal or physeal line or end of ulna
2	Midline point of epiphyseal or physeal line or end of radius
3	Proximal side angle point of hamate bone
4	Proximal side outer margin point of capitate bone
5	Ulnar side outer margin point of hamate bone
6	Radial side outer margin point of hamate bone
7	Ulnar side outer margin point of capitate bone
8	Radial side outer margin point of capitate bone
9	Distal midmargin point of hamate bone
10	Distal midmargin point of capitate bone
11	Midline point of proximal side 5.metacarpal bone
12	Midline point of proximal side 4.metacarpal bone
13	Midline point of proximal side 3.metacarpal bone
14	Midline point of proximal side 2.metacarpal bone
15	Midline point of 1. proximal side metacarpal end or epiphyseal or physeal line
16	Midline point of 5. distal side metacarpal end or epiphyseal or physeal line
17	Midline point of 4. distal side metacarpal end or epiphyseal or physeal line
18	Midline point of 3. distal side metacarpal end or epiphyseal or physeal line
19	Midline point of 2. distal side metacarpal end or epiphyseal or physeal line
20	Midline point of distal side 1. metacarpal bone

### 2.5. Geometric morphometric analysis

Procrustes analysis was applied for the comparison of shape differences and evaluation of growth and allometry. Homogeneity of the covariance-variance matrices was examined with Box’s M test. Since the covariance-variance matrices were not homogeneous, James’ FJ test was used for shape comparisons. To obtain an overall measure of shape variability, the root mean square of Kendall’s Riemannian distance rho to the mean shape was taken into consideration.

The shape deformations were assessed using thin plate spline (TPS) analysis. Procrustes shape means were calculated for TPS analysis. Based on the TPS analysis results, the areas showing the greatest reductions or enlargements were marked in different colours in order to indicate deformations. The relationships between the centroid sizes and bone age were examined by the growth curve models. The assessment of the fit of the models was made based on the coefficient of determination (R2). The allometry assessment was made with multivariate regression analysis of the centroid size and tangent coordinates. The assessment of the fit of the models was based on the mean square error (MSE). The significance of the model was examined with the Wilks’s lambda test. Shape changes for different centroid sizes were examined with a multivariate regression model. In this study, Rv3.3.2, PAST v3.20 and NCSS v07.1.5 software was used for statistical analysis.

### 2.6. Landmark reliability

The intrarater reliability coefficient was calculated for a two-facet crossed design (‘landmark pairs-by-rater-by-subject’, l × r × s) based on generalisability theory (GT) [17]. In GT, the reliability of relative (norm-referenced) interpretations is known as the generalisability (G) coefficient [18]. In this study, the landmarks were marked by the same investigator. After a month, the same researcher marked the landmarks on 20 radiographs of hands and wrists selected randomly from the study population. An analysis was made to obtain a G reliability coefficient. The rating indicated a strong repeatability for subjects (G = 0.9989).

## 3. Results

**Figure 2:**
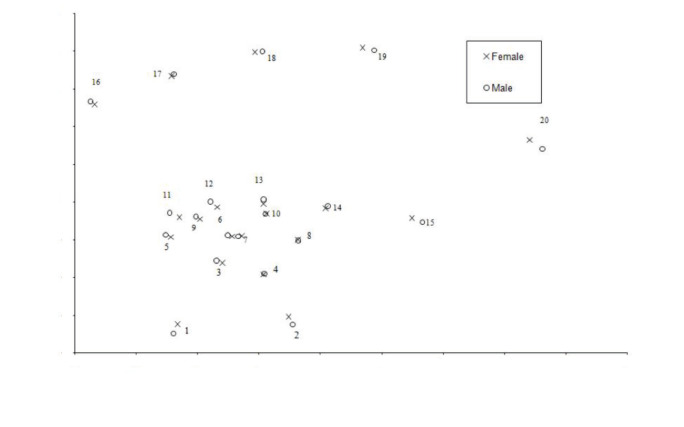
Procrustes mean shape for hand and wrist landmarks (1-20) images of male (o) and female (x).

Shape comparison of the hand and wrist between the sexes was performed by using statistical shape analysis. Statistically significant differences were found in terms of general hand and wrist shapes between the sexes (P = 0.009). Figure 2 shows the Procrustes mean shape of the hand and wrist according to sexes. The general shape variability of the hand and wrist was found to be in 0.084 for females and 0.086 for males.

**Figure 3:**
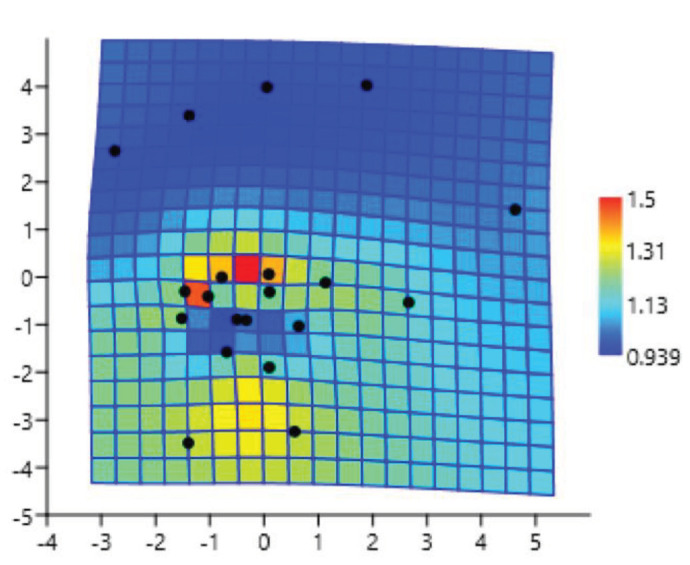
Thin plate spline graphics demonstrating shape deformation from female to male. Expansion factors at the landmarks are greater than one.

Procrustes mean shapes were calculated for TPS (Figure 3). In accordance with the TPS method, the points displaying the greatest enlargement and shrinkage were labelled as deformations. Expansion factors at the landmarks are shown coloured (expansion factors greater than one) (Figure 3). Deformations were seen in the shape of hand and wrist according to sex.

The highest deformation was seen at the midline point of the proximal side of the 3rd and 4th metacarpal bones (landmarks 12-13), the distal midmargin point of the hamate and capitate bones, the midline point of the proximal side of the 5th metacarpal bone (landmarks 9-11), the midline point of the epiphyseal or physeal line or end of the ulna (landmark 1), the midline point of the epiphyseal or physeal line or end of the radius (landmark 2), and the proximal side of the outer margin point of the capitate bone (landmark 4), respectively.

### 3.1. Growth evaluation

**Figure 4:**
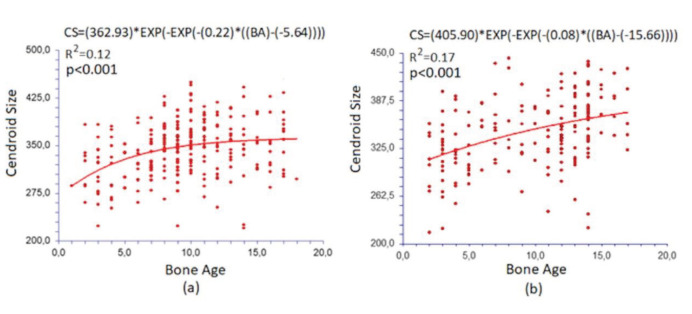
Growth curve between centroid size (CS) and bone age (BA) in females (a) and males (b).

The most appropriate model in the growth models was seen as the Gompertz growth model for both females and males (Figure 4).

When the age-related change of centroid size was examined, it was observed that the growth in females, which increased regularly until the age of about 10, became stable after the age of about 10, whereas there was an ongoing growth curve in males (Figures 4a and 4b).

### 3.2. Allometry evaluation

**Figure 5:**
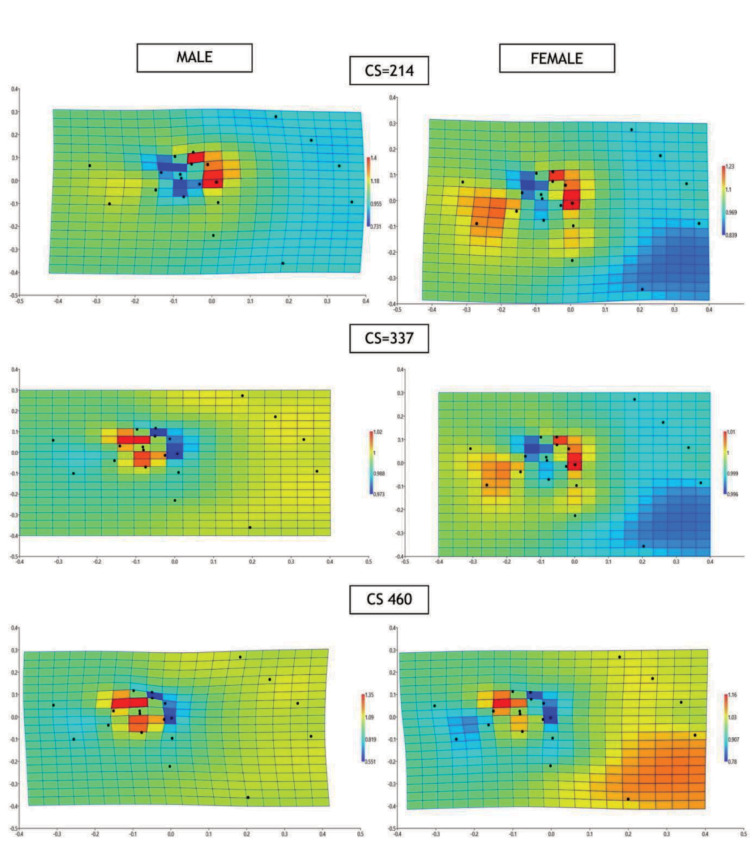
The horizontally oriented TPS graphs (Landmarks 1-2 are on the left side- landmarks 16-17-18-19 are on the right side, landmarks 15-20 are at the bottom side) were formed according to the lowest centroid size (CS) value observed of 214, the mid CS value of 337 and the highest CS value of 460 to see the effect of growth on shape in male and female. Expansion factors at the landmarks are greater than one.

As a result of the multivariate analysis applied for the relationship between size and shape to assess allometry, significant models were obtained in females (model P = 0.017, MSE = 0.0002) and in males (model P < 0.001, MSE = 0.0002). Shape changes associated with size in males and females are presented as TPS graphs. Centroid size is a nonsize specific unit size. Centroid size is a composite measure of size based on all landmarks. In our study, after calculating the centroid size of the units having the smallest and largest centroid size values, the average of these two values ​​was taken as the median centroid size, and the TPS graph obtained from the allometric model created to examine the relationship between growth and shape was examined. The TPS graphs were formed according to the lowest centroid size (CS) value observed of 214, the median CS value of 337 and the highest CS value of 460 to see the effect of growth on shape (Figure 5).

In our study, allometry was evaluated in three stages considering the centroid size criteria in our data set. When the relationship between size and shape was evaluated, it was observed that the areas where deformation occurred with males varied; on the other hand, it was found out that shape deformations in females were more stable (seen in similar areas). In the first stage of growth (CS = 214), a similar allometric structure was observed in females and males, but an additional morphological change was observed in the radiocarpal area (region of landmarks 1-2-4). In the second stage of growth (CS = 337), it was observed that the shape change was in the same areas in females, whereas the area where the shape variability was observed in the growth of men had changed from distal (landmarks 9-10-11-12-13) to proximal (landmarks 3-5-6-7-8). In the third stage of growth (CS = 460), it was observed that there was a change in shape in similar areas compared to the previous growth stage in males, whereas it was observed that the areas in which shape change was observed in females differed. It was observed that the shape change from distal to proximal in females was in the last stage of growth.

## 4. Discussion

Statistical shape analysis is necessary to quantify the variations in shape across a population. Furthermore, statistical shape analysis makes it possible to examine specific anatomical regions and investigate local shape changes. In the current study, statistical shape analysis was performed in order to evaluate the changes in the shapes of the hand and wrist between sexes and the correlation between growth and allometry.

The parameters used in bone age classification are the time of the first visualisation of ossification centres, distribution in the ossification process and union with each other of bone parts [19]. The carpal bones are not ossified at birth, and become ossified through primary ossification centres and then grow [20]. Longitudinal growth in the long bones is seen with primary and secondary ossification centres [19]. When there is distal growth cartilage in the metacarpal bones, this refers to the proximal part of the thumb. In the longitudinal growth of the bones, continuous production of growth cartilage has a role in the ossification of old cells. When growth reaches an end, the cartilage is covered. A line is seen on radiographs. The ossification centres of the capitatum and hamatum are seen in the 2nd–4th months, and of the pisiform bone in the 9th–12th months [21]. The ossification centres of the metacarpal bones start to be differentiated at 10–12 months in females and at 14 months–3 years in males [22]. In the current study, landmarks were defined as anatomical sections (distal radius and ulna with metacarpals) and ossification centres (hamatum and capitatum) starting to be seen in the early months in all age groups.

Sex and ethnic background are an expected outcome for skeletal maturation, as are hand and wrist shape [23–25]. The effects of sex on hand/wrist shape and sex-related differences were studied using different methods [23–25]. Several studies have found that there is significant difference between the hand bones of males and females [23–25]. Crisco et al. reported in their study that although carpal bones in females were significantly smaller than carpal bones in males, individual carpal volume as a percentage of entire carpal volume did not differ according to sex [23] *.* Schneider et al. found that size was the only difference between men and women in morphology of the trapezia and first metacarpal bones [24]. These findings confirm their initial hypothesis, and the fact that across the population, women have similarly shaped trapezia and first metacarpals compared to men is important in understanding functional anatomy and pathology of the thumb [24] *.* Didi et al. emphasised that carpal bones in males were of higher volume than carpal bones in females [25]. Besides, Didi et al. reported that all 8 carpal bones exhibited varying degrees of sexual dimorphism [25] *.* In our study, the Procrustes mean shapes were calculated and the shape deformations of the hand and wrist were assessed using thin plate spline (TPS) analysis. It was found that there was a statistically significant difference in hand and wrist shape in terms of sex. In our study, it was found that the highest deformations were determined at the midline point of the proximal side of the 3rd and 4th metacarpal bones, the distal midmargin point of the hamate and capitate bones, the midline point of the proximal side of the 5th metacarpal bone, the midline point of the epiphyseal or physeal line or end of the ulna, the midline point of the epiphyseal or physeal line or end of the radius, and the proximal side of the outer margin point of the capitate bone.

Sex differences in the speed of growth and development, the timing of the growth spurt in adolescence and the skeletal maturation age are well known. The characteristics of sexual maturation, chronological age, weight, height, dental development and skeletal development are among the more common means used to identify stages of growth. It is extremely important to determine maturation and evaluate subsequent growth potential during preadolescence or adolescence. Another important aspect in the determination of bone age is the determination of adult height and the level of bone age as a result of various diseases [26]. There are growth percentile curves according to sex, weight, height and age [27]. Bone age in females is advanced at every age compared to males, and this difference is a little more evident after the onset of puberty, and thus the skeletal maturation of males lasts longer than that of females [28,29]. Epiphyseal fusion occurs approximately 2 years earlier in females than in males [1]. Hägg U and Taranger J. found that all skeletal stages and growth events occurred at earlier ages in females than in males [30]. In addition, they found in their study that at the peak of the adolescent growth spurt, skeletal development was more advanced in females compared to males, but that at the end of the spurt, females had a less mature skeletal development than males [30] *.* The assumption that the appropriate measurement of size is the size of the anatomical structure analysed, usually evaluated as the centroid size of a landmark configuration, is encountered in many morphometric analyses [10–12,16] *.* Centroid size is a composite measure of size based on all the landmarks and is proportional to the square root of the summed square interlandmark distances. It has been shown not to be correlated with shape for small isotropic variations at each landmark [31,32]. We examined the relationship between skeletal age (bone age) and centroid size, calculated using geometric morphometric methods. In our study, when the age-related change of centroid size was examined, it was observed that the growth in females, which increased regularly until the age of about 10, became stable after the age of about 10, whereas there was an ongoing growth curve in males. In the current study, bone age and shape size were examined with growth curves according to male and female sex. When growth was evaluated up to 18 years on these graphs, rapid growth was seen in females up to the middle years of childhood, followed by a decrease in rapid growth. In males, however, there occurred a continuous increase in growth at the same rate.

In their study of sex-related shape variation and allometric pattern in the carpal bones making up the radiocarpal and midcarpal joints in modern human beings, Kivell et al. found that many features of carpal shape (76% of all variables evaluated) were similar between females and males, despite variation in size [33]. However, in their study, there was some aspect of shape in each carpal bone that was significantly sexually dimorphic. In total, 10 shape ratios (24%) significantly differed between females and males [33]. Kondo et al. examined the variance models in hand ratios based on principal component analysis [34]. Their results demonstrated that males have significantly longer metacarpals and phalanges than females [34]. Furthermore, there is a significant difference in hand proportion between males and females, with males having relatively longer distal phalanges among finger bones [34]. Also, males have relatively shorter second but relatively longer fourth fingers than females in their study [34]. In another study, Kondo et al. found that the proximal bones scaled with comparatively smaller allometry coefficients than the distal bones in the human hand [35]. The change from distal to proximal is consistent with the fact that the two ossifications start with the capitate bone, one of the distal row carpal bones. They reported that no statistical differences were observed in the allometric relationship between males and females for all the examined hand bones except the 5th middle phalanx, possibly owing to larger variabilities in female 5th middle phalanx length [35]. In addition to the understanding of secular change in human hand ratios, they emphasised that their results will contribute to normal growth and development models [35]. In our study, allometry was evaluated in three stages according to the centroid size criteria in our data set. In the first stage of growth (CS = 214), a similar allometric structure was observed in males and females but an additional morphological change was observed in the radiocarpal area (region of landmarks 1-2-4). The difference between the sexes in the radiocarpal region due to growth (allometric variation) supports the thesis of Kivell et al. [33]. When the effect of size on shape was evaluated on the allometry graphs using centroid size, it was observed that there was a change in the females and males in the distal to proximal with the growth, i.e. from the carpometacarpal area to the carpal bone region. According to centroid size analyses, moreover, shape variation from distal row carpal bones to proximal row carpal bones was determined. The change emerging in this direction started earlier in males and later in females, but the areas where the change occurred were similar. In their article, Kivell et al. stated that if there is a sex difference in the wrist bones, if there is a formal difference, this should be the case for the radiocarpal joint and midcarpal joint which account for more than half the range of motion of the wrist [33]. Besides, sex differences in carpal kinematics have been found in previous studies, and there are also discrepancies in the location of the flexion/extension and the radio-ulnar deviation and rotation axes of the wrist in the literature [36] *.* It has been suggested that these differences are due to differences in carpal bone size rather than in sex, and they can be resolved by normalising the kinematics by carpal size [36] *.* The morphometric features of carpal bones such as shape and curvature are closely associated with the resulting mechanics of the related joint. In our study, in the first stage of growth (CS = 214), a similar allometric structure was observed in females and males, but an additional morphological change was observed in the radiocarpal area (region of landmarks 1-2-4) in females. Characterising the morphology of the carpometacarpal, midcarpal and radiocarpal joint bones and how they differ across the population is important in order to understand the functional anatomy and pathology of the hand and wrist. Are the growth characteristics of the wrist bones important in terms of pathologies such as osteoarthritis and carpal tunnel syndrome and sex effect on growth-related deformation? Also, osteoarthritis and carpal tunnel syndrome is known to be more common in female and the question of whether the morphological features of the wrist bones are a risk factor in this syndrome should be investigated in further studies on the basis of this article.

We have presented a growth model for hand and wrist bones with this study. We have examined the effect of bone maturation (bone age = growth) on the shape of the bones. We have also revealed sex differences in the shape change. Moreover, we have examined the effect of growth on the shape of the bones. In our study, the difference between the sexes was found mostly in the radiocarpal region. The ossification properties and procedures of the skeleton are related to changes in size and shape. Consistent with this situation, in our study, it was found that the shape deformation of the carpal bones first began in the distal rows of the carpal bones.

There were some limitations to this study. The cases included in the study were those who had been presented at the Paediatric Endocrinology outpatient clinic for various reasons and had had left-hand wrist radiographs taken. Therefore, the results may not fully reflect healthy individuals. On  the other hand, this study can be considered of value in respect of its potential for guidance in further studies conducted with patients diagnosed with specific diseases and/or completely healthy cases.

In conclusion, to the best of our knowledge this is the first study in the literature to have been done for shape comparison, growth and allometry analysis in a structure examination with the geometric morphometric analysis method on hand wrist radiographs. Significant differences were found in the hand and wrist shapes according to sex. Models for growth and allometry of hand and wrist shapes were found to be significant in children. 

## Financial disclosure

The authors declared that this study has received no financial support.

## Informed consent

Approval for the study was given by the Local Ethics Committee (2017/13/08) and all procedures were applied in conformity with the Declaration of Helsinki. We waived the informed consent due to the retrospective nature of the study.
